# Targeted Molecular Imaging in Oncology

**DOI:** 10.1038/sj.bjc.6600300

**Published:** 2002-05-06

**Authors:** C Brock

**Affiliations:** Royal Marsden Hospital, Sutton, UK

## Abstract

*British Journal of Cancer* (2002) **86**, 1528–1528. DOI: 10.1038/sj/bjc/6600300 www.bjcancer.com

© 2002 Cancer Research UK

## 

E Edmund Kim and David J Yang. Publishers: Springer-Verlag, 2001. ISBN 0-387-95028-1. £120.50, $169.00.

As the foreword to this book states, our passage into the new millennium gives the opportunity to use the medical achievements of the past 1000 years to develop treatment strategies for the future. It describes and explains the roles that imaging plays in the emergence of new diagnostic and therapeutic procedures.

Increased knowledge regarding the genetics and the molecular biology of tumour development offers the potential of new targets of therapy. However, devising methods with which to determine the existence, and the evaluation, of novel diagnostic and therapeutic strategies in cancer remains a challenge. Improved imaging techniques and the radiolabelling of tracers and drugs are making non-invasive imaging of molecular targets possible, thereby facilitating the translation of these scientific breakthroughs into clinical practice.

This book provides an excellent review of imaging technologies and their role within oncological treatment strategies. It begins with a basic science chapter giving a very structured and readable background to the molecular genetics of cancer, cancer cell immunology, biochemistry and pathology. This chapter explains DNA replication and the genetic mutations that give rise to the activation of oncogenes and the inactivation of tumour suppressor genes in neoplasia. It discusses the role of cellular immunology, including T-cell and B-cell immunity and cytokine interactions, providing an insight into active and passive immunotherapy in the treatment of cancer. This gives the reader a good platform from which to proceed with the subject of molecular targeting.

The following chapters cover the principles of each of the imaging technologies available, and include the techniques of ultrasound, computerised tomography (CT), magnetic resonance imaging (MRI) and spectroscopy (MRS), single positron emission tomography (SPECT) and positron emission tomography (PET). Each chapter addresses the difficult topic of technique methodology and the authors manage to describe this in a surprisingly straightforward manner. Obviously, some chapters are slightly more complicated than others, but in general the points are made clearly and simply.

Having set the scene and discussed the various advantages and disadvantages of each imaging method, the second part of the book gives examples of the ways in which they can be used to image molecular targets utilising, for instance, radiolabelled antibodies and radiopharmaceuticals. Chapter 5 provides an overview of some of the radiopharmaceuticals that target tumours, concentrating on the rationale and the results of these studies.

The radiolabelled antibody chapter, Chapter 6, is particularly interesting. It covers the production of monoclonal antibodies from hybridomas, made from the fusion of myeloma cells and B-lymphocytes, through to current phage library techniques. The importance of optimising antibody selection for tumour targeting, in particular the kinetics according to fragment size, is highlighted. Finally, the radioisotopes available for antibody radiolabelling are discussed. The following chapter expands upon this theme by demonstrating the possibility of radioimmunodetection of different tumour-marker expressing cancers using commercially available monoclonal antibody conjugates, such as ProstaScint (Cytogen) for prostate carcinoma, CEA-Scan (Immunomedics) for colorectal cancer and OncoScint (Cytogen) for colorectal and ovarian cancer.

The potential value of polymer-drug conjugates and macromolecular contrast media imaged with MRI are described, together with the use of paramagnetic radionuclides to label monoclonal antibodies, and newer contrast agents, to aid in specifically targeting these for magnetic resonance imaging. This, thereby, expands the use from gadolinium-based interstitial agents to agents tailored to specific clinical applications.

Dynamic PET images have been likened to displaying ‘slices of life’, however the expense of performing these scans generally confines their use to academic and teaching medical centres. On the other hand, SPECT is more accessible. Both techniques provide 3-dimensional images of the distribution of radiotracers and the chapters regarding targeted SPECT and PET provide data and images of gallium-67, thallium-201, ^99m^technetium-sestamibi scans in SPECT and fluorine-18 fluorodeoxyglucose scanning in PET. The main thrust of these chapters is to describe those radiotracers with established roles in oncology, with suggestions of those requiring further investigation.

The final third of the book expands upon the roles of the different techniques for imaging the processes important in tumour development and survival, in particular angiogenesis, hypoxia, apoptosis and signal transduction. The potential for imaging anti-cancer drugs themselves, including anti-sense oligonucleotides, gene delivery and expression, is also discussed. Illustrations are shown and the images and results are discussed providing a vast array of potential and future applications for targeting imaging.

Overall, this is a very informative, readable book. The chapter sizes are easily digestible and there is a clear progression through the book presenting a succinct and balanced picture of the methodologies available. The black and white images of the techniques and the targets help to enlighten the reader visually regarding the text. I thoroughly enjoyed reading the book and learning from it. I would recommend it to both the reader with some knowledge wishing to explore the topic further as well as to those wanting a basic introduction and background to the subject.

